# Swedish massage as an adjunct approach to Help suppOrt individuals Pregnant after Experiencing a prior Stillbirth (HOPES): a convergent parallel mixed-methods single-arm feasibility trial protocol

**DOI:** 10.1186/s40814-024-01499-z

**Published:** 2024-04-30

**Authors:** Sarah Fogarty, Alexander E. P. Heazell, Niki Munk, Phillipa Hay

**Affiliations:** 1 School of Medicine, Western Sydney University, Locked Bag 1797, Penrith, NSW 2751 Australia; 2 Translational Health Research Institute, Western Sydney University, Locked Bag 1797, Penrith, NSW 2751 Australia; 3 School of Medical Sciences, Maternal and Fetal Health Research Centre, University of Manchester, Manchester, UK; 4 Department of Obstetrics, Saint Mary’s Hospital, Manchester University NHS Foundation Trust, Manchester, UK; 5 School of Health & Human Sciences, Indiana University, Indianapolis, USA; 6 Australian Research Centre in Complementary and Integrative Medicine (ARCCIM), Fellow and Visiting Faculty of Health, University of Technology Sydney, Massage & MyotherapyAustralia, Sydney, Australia; 7 Mental Health Services, SWSLHD, Campbelltown Hospital, Campbelltown, NSW Australia

**Keywords:** Massage, Stillbirth, Mixed-methods, Termination for medical reasons, Pregnancy after loss

## Abstract

**Background:**

Women experiencing pregnancy after stillbirth experience high levels of anxiety, fear and depression. Standard antenatal care may be emotionally unsuitable for many women at this time, and there is a lack of evidence on what interventions or approaches to care might benefit these women. Therapeutic massage may assist women after stillbirth by decreasing anxiety, worry and stress.

**Objective:**

This paper outlines the objectives, methodology, outcome and assessment measures for the *Helping suppOrt individuals Pregnant after Experiencing a Stillbirth* (HOPES) feasibility trial which evaluates massage as an adjunct approach to care for pregnant women who have experienced a prior stillbirth. It also outlines data collection timing and considerations for analysing the data.

**Methods:**

HOPES will use a convergent parallel mixed-methods, single-arm repeated measures trial design in trained massage therapists’ private clinics across Australia. HOPES aims to recruit 75 individuals pregnant after a previous stillbirth. The intervention is massage therapy treatments, and participants will receive up to five massages within a 4-month period at intervals of their choosing. Primary quantitative outcomes are the feasibility and acceptability of the massage intervention. Secondary outcomes include determining the optimal timing of massage therapy delivery and the collection of measures for anxiety, worry, stress and self-management. A thematic analysis of women’s experiences undertaking the intervention will also be conducted. A narrative and joint display approach to integrate mixed-methods data is planned.

**Discussion:**

The HOPES study will determine the feasibility and preliminary evidence for massage therapy as an intervention to support women who are pregnant after a stillbirth.

Trial registration.

ClinicalTrials.gov NCT05636553. Registered on December 3, 2022, and the trial is ongoing.

## Background

There are almost 6 stillbirths a day in Australia (1, 2) where stillbirth is defined as ‘in-utero death from a 20-week gestation until immediately before birth’ (3). Stillbirth profoundly alters the reality of subsequent pregnancies (4). Pregnancy after stillbirth has been described by mothers as stress that is ‘survived’. (5) The impact of stillbirth on subsequent pregnancies is profound with mothers commonly describing conflicted emotions, high levels of anxiety and stress, fear, isolation, and a lack of trust in a good outcome (5–8). Women who have experienced a stillbirth in their previous pregnancy are at increased risk of adverse pregnancy outcomes in subsequent pregnancies such as pre-term birth and low birthweight (7, 9). The mental health of women experiencing pregnancy following stillbirth is similarly affected; with women experiencing post-traumatic stress disorder symptoms (10) and significantly more depression and anxiety than pregnant women who had not experienced a stillbirth (11, 12). Standard antenatal care is emotionally unsuitable for many women in pregnancies following a stillbirth due to their increased need of psychosocial support (13). Negative experiences of standard antenatal care include exacerbated stress and reawakened traumatic memories (5). There are few specific specialist antenatal services for women to access in a subsequent pregnancy after a stillbirth (5, 14). The international stillbirth research community has highlighted concerns about the use of traditional randomised control trial (RCT) methodologies especially when evaluating psychosocial support interventions in a pregnancy following stillbirth (15). Specific ethical concerns raised include the constraints of having a ‘treatment as usual/standard care’ intervention and withholding potentially beneficial care from those who need it (15). Methodological constraints include large sample size requirements, particularly in studies where all groups receive the active intervention (16, 17) and ‘RCT methodologies being incompatible with a much-needed individualised approach to care’ (15). Evaluation concerns in this population have contributed to the lack of direct evidence on what specific interventions or approaches to care might benefit women experiencing pregnancy after stillbirth including adjunct interventions (15). More research is needed on interventions that benefit mothers and improve their emotional health during a pregnancy after stillbirth.

Pregnancy massage aims to support the physiologic, structural and emotional well-being of both mother and fetus using various massage techniques including the Swedish and remedial massage. There is growing evidence that massage can benefit emotional health in non-pregnant and pregnant populations in various ways including by decreasing anxiety (18–25), lowering stress (20, 22, 24, 26, 27), decreasing depression (19–23, 25, 28–30) and improving mood (22, 24, 25, 31, 32). Qualitative data for massage and bereavement found massage to be helpful for recipients by generating feelings of consolidation and cultivating the feeling of support and care (33, 34). There is limited research on the aggregate effects of massage care during therapeutic alliance building, one-on-one care over time and when individualised treatments are part of the ‘treatment’ alongside massage techniques or strokes (35). A recent case study found that a patient-centred massage treatment can be a support option for women experiencing a pregnancy after stillbirth with massage assisting the ability to cope during that stressful time (36).

Massage therapy is more than the application of a massage technique and is a philosophy of care including many non-manual factors; more research encompassing massage as a philosophy of care, particularly in the antepartum population, is needed. Massage has demonstrated a precursory capacity to address the somatic and psychological symptoms associated with being pregnant after a stillbirth as well as some of the altered psycho-behavioural factors that occur in a clinical context (36). Given the precursory evidence for massage as a support option for women pregnant after stillbirth and the highlighted ethical and methodological concerns regarding withholding potentially beneficial care, treatment as usual intervention groups, sample size requirements and the need for individualised treatments for this population, a study focused specifically on the feasibility of a massage therapy intervention trial in this population is warranted before embarking on larger and more expensive effectiveness research. This paper outlines the objectives, methodology, outcome and assessment measures for the Helping suppOrt individuals Pregnant after Experiencing a Stillbirth (HOPES) feasibility trial which evaluates individualised Swedish massage as an adjunct approach to care for pregnant women who have experienced a prior stillbirth.

### Objectives

The specific objectives of the study are as follows:

1. Assess the feasibility and acceptability of the massage intervention in the pregnant population who have a prior stillbirth experience;
2. Determine the optimal timing of therapy, and measurement collection needs for anxiety, worry, stress and self-management;
3. Conduct a thematic analysis of women’s experiences undertaking the intervention to evaluate the acceptability of the study processes and the intervention.

## Method

The HOPES feasibility trail began enrolment in February 2023 and expects to complete data collection in mid-2024.

The HOPES study utilizes a convergent parallel mixed-methods, single-arm repeated measures trial design aiming to assess the feasibility of Swedish massage as an adjunct approach to care for pregnant women who have previously experienced stillbirth. The convergent parallel mixed-methods design collects and analyses quantitative and qualitative individually, then compares and relates the two data types for areas of convergence or divergence and then interprets the meaning of the combined results. The primary quantitative outcomes are the feasibility and acceptability of the massage intervention. Secondary outcomes include determining the optimal timing of massage treatments and the collection of measures for anxiety, worry, stress and self-management. A thematic analysis of women’s experiences from a subsample of participants undertaking the intervention will also be conducted. The study intervention consists of up to five 60-min pregnancy massage treatments over a 4-month period at individualized time intervals per participant’s choice or availability. Each participant will complete outcome measures pre- and post-study intervention. Participants will complete a short outcome measure each pre- and post-massage treatment. A narrative and joint display approach to integrate mixed-methods data is planned.

### Study design

The study is a convergent parallel mixed-methods, single-arm repeated measures pilot trial design.

### Ethics approval and consent to participate

Human ethics approval for the study was granted by the Ethics Committee of Western Sydney University (No: H15261). Changes in protocol will undergo ethical approval. All study participants will receive written information about the study with the option for information to be provided orally. All study participants will give informed written consent for the use of their data and the application of the intervention. Participation in the study is voluntary and can be refused.

Data are treated confidentially and processed pseudonymously. The collection and storage of personal data take place in accordance with the University’s Research Data Management Policy. Shared information collected from the participants is non-identifiable using a study identifier (number). Quantitative data collected will be available for use in any other research projects in the future. To make reuse of the quantitative data possible, it will be stored under Western Sydney University’s Open Access Policy.

### Recruitment and screening

Potential participants are recruited via social media and from maternity clinics, shared care General Practitioners and obstetrician clinics around the massage therapists’ locations within Australia. Maternity services and clinics approached are based on the proximity of services to the locations of the massage therapists participating in the study. Potential participants are informed about the study via their obstetric healthcare provider or via social media platforms for stillbirth support services such as SANDS (37) and Red Nose (38). Potential participants contact the researchers via the shared contact information who explain the study and screen potential participants for eligibility (see 2.4.1 and Table 1). Potential participants are given a clear explanation of the requirements of the study, the opportunity to ask questions and time to consider their participation. Individuals who agree to take part must sign an informed consent form. Consenting individuals meeting the criteria of the study are then enrolled in the study.

**Table 1:** Inclusion and exclusion criteria

Inclusion criteria
● Pregnant individuals 18 years of age or greater who have experienced a stillbirth or termination for medical reasons (TFMR) from 20 weeks of gestation in a previous pregnancy
● Participants must be less than 30 weeks of gestation
● Participants must be able to attend intervention visits at one of the study pregnancy massage therapists’ clinics located within Australia
● Be able to complete the intervention visits within in a 4-month period
Non-inclusion criteria
● Potential participants will be excluded if their schedule will not allow them to receive the study treatments in the allocated time frame

### Eligibility criteria

A purposeful sample of pregnant women are being recruited for the HOPES study who have experienced a stillbirth or termination for medical reasons (TFMR) after 20 weeks of gestation in a previous pregnancy (see Table 1). To be eligible to participate, pregnant women need to be less than 30 weeks of gestation. There are no criteria for eligibility related to the interpregnancy interval between the stillbirth and the current pregnancy because evidence shows the impact of stillbirth can be felt for years, and sometimes decades, after the loss (8, 39–41). The mental health of participants experiencing pregnancy after a stillbirth is varied, and we chose not to limit access to the study based on only one or two of the many mental health symptom participants may experience (e.g., anxiety, stress); especially as the mental health of participants is expected to be impacted at different time points during the pregnancy and be unique to each individual and situation (39).

### Sample size

The study aims to recruit a total of 75 pregnant people meeting the study criteria. This feasibility study is not expecting large effect sizes as anxiety and depression experienced during a pregnancy after stillbirth do not decrease until 6 months post birth (42). Thus, it is hypothesized that while the immediate effects of the massage intervention might be larger, the long-term effects will likely be small (< 0.1). Using the approach by Whitehead et al. for small effect sizes (43), it is estimated a minimum of 50 pregnant people is needed to reliably detect a small effect size (< 0.1). A sample of 75 pregnant women allows for a 10–15% drop out rate and 10% for whom data is incomplete.

A sub-group sample of 20 participants will be invited via purposive sampling to participate in the qualitative phase of the mixed-methods design. Twenty participants have been proposed as optimal to ‘improve open and frank exchange of information and mitigate some of the bias and validity concerns inherent in qualitative research’ (44). Purposive sampling will consider the type of loss (stillbirth and TFMR), early stillbirth/TMFR loss (losses between 20 and 29 + 6 weeks of gestation) and late stillbirth/TMFR loss (losses between 30 and birth).

### Intervention

**Fig. 1:**
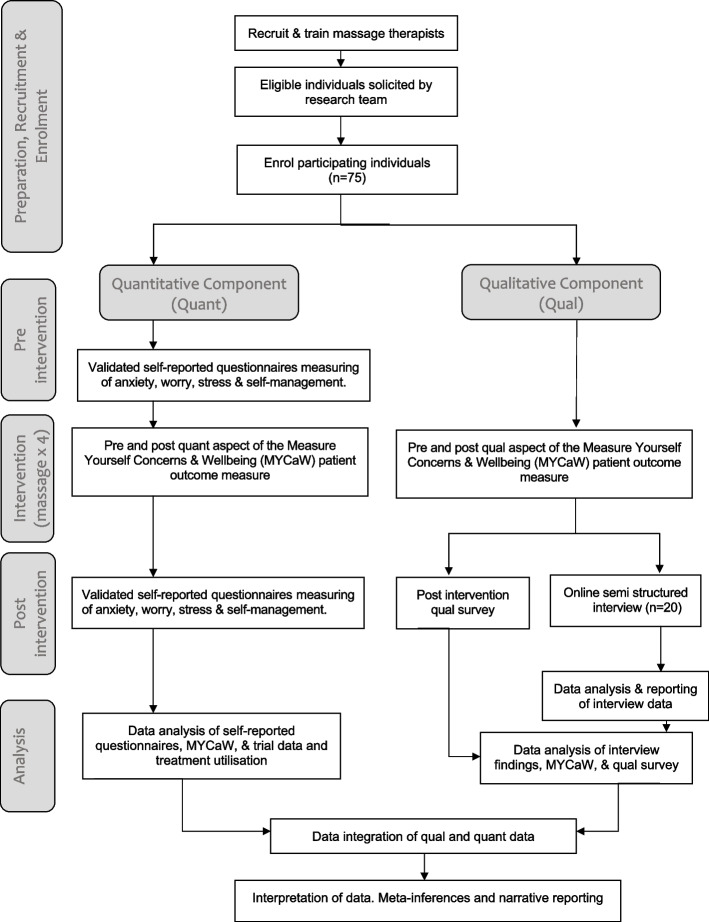
CONSORT diagram of participant flow through the study

Each participant will have access to up to 5 allocated 60-min massage consultations see Fig. 1. The massage treatments will be administered within a 4-month period at intervals of participant and therapist dyad determining based on participant preference and therapist treatment planning. Each massage consultation will be individualised to meet the needs of the participant on the day of treatment. The conceptual approach utilized is based on a vulnerability-to-stress concept. The vulnerability-to-stress concept acknowledges the impact of stress on vulnerability and recognises protective factors that can help reduce stress (45). For women pregnant after a stillbirth, the vulnerability is the previous loss (and any other pregnancy losses) as well as any biological factors, and the stress is being pregnant again (and the fear of another loss). We are proposing that massage might be a protective factor to help manage stress. This conceptual approach was used in a published case study (36). The massage aims to support women by addressing altered psycho-behavioural and physiological factors associated with vulnerability and stress such as countering feelings of loss of control via allowing participants to individualise their treatments (e.g. depth, area of the body to be treated, what they want to be treated). See Additional file 1 for the protocol for the massage which covers more in-depth how massage may do this. The massage intervention uses hands-on techniques, listening and creating the environment to build a therapeutic alliance to help provide protective factors and manage the stress of pregnancy after a stillbirth. Allowable massage strokes for therapist incorporation into treatment to address the study aims include longitudinal gliding, transverse gliding, digital ischemic pressure, transverse frictions and transverse gliding. The use of these massage strokes is based on prior pregnancy massage research (39, 42). The areas of the body treated, the depth of treatment and which massage strokes used will be determined by the massage therapist reflecting real-world clinical massage practice. No other form of massage therapy or hands-on modalities (e.g. reflexology, aromatherapy massage) are to be used and nor are other healing modalities (e.g. reiki). Treatment sessions are 60 min and include hands-on treatment time as well as the time prior and after the hands-on massage to consult with the participant about their treatment and plan the timing for the next treatment session. See Additional file 1.

### Delivery of the intervention

The massages will be provided by a team of massage therapists with experience in pregnancy massage and perinatal loss across multiple sites within Australia. Massage therapists will be recruited (1) through the study personnel who have previously worked with the therapists and or (2) through an expression of interest via a pregnancy massage training organisation in Australia. Therapists from all states and territories across Australia who had completed pregnancy massage training post-completion of their initial massage qualifications are able to express an interest in being study massage therapists. At least 20 massage therapists will be recruited to ensure that there are therapists in differing states and territories across Australia due to the difficulty of recruiting 75 people from a more limited geographic area. The exact number of therapists to be recruited is unknown as it is undetermined how many therapists will express an interest. The massage therapists attend an online 3-h training on the massage study intervention and facilitation of the massage experience for individuals pregnant after a loss as well as training on grief and resources available to individuals pregnant after a loss prior to becoming study massage therapists. The massage treatments are administered in the massage clinician’s office and follow a protocol that allows for treatment to be tailored for each participant depending on the presentation on the day. See Additional file 1 for massage protocol. The focus of each treatment is determined by the participant via the Measure Yourself Concerns and Wellbeing (MYCaW) patient-reported outcome measure (PROM) (46), and the massage therapist administers remedial and or relaxation massage techniques.

### Side effects

Participants will be sent a questionnaire 24 to 48 h after each massage treatment asking about experienced side-effects from the massage treatment such as post-massage soreness, headaches, tiredness or any other side effects (please specify). These side effects are based on research into side effects, massage and pregnancy (47). Adverse events are recorded and reported quarterly to the Research Ethics Committee, and serious adverse events are required to be reported immediately (Additional file 2).

### Criteria for discontinuing the intervention

The intervention will cease if participants are advised by their obstetric healthcare providers (i.e. on bed rest), if they are no longer pregnant or if the study participant delivers their baby prior to completing the allocated study intervention.

### Assessments

The measures used to provide data to meet the three objectives of the study are as follows:

#### Trial data

Data will be collected by the study team throughout the trial on the number of inquiries, number of participants, drop-out rates and compliance with completing the validated outcome measures. See Table 2.

**Table 2:** Outcome measures and timing

Project outcome	Measured by^a^	Timepoint				
*To assess the feasibility and acceptability of the massage intervention including intervention delivery and outcome measure compliance*						
		At enrolment	Post recruitment completion	*Study intervention*	Post-study intervention completion	At end of the study
				At each study treatment visit		
Feasibility	Number of enquiries		✓			
	Recruitment rates					✓
	Retention rates					✓
	Compliance with completing the outcome measures					✓
Acceptability						✓
○ Acceptability of the study processes	Retention rates					✓
	Response quality/compliance with the validated outcome measures					
	Post-intervention qualitative questionnaire				✓	
	In-depth interviews with study participants				✓	
○ Acceptability of the study intervention (massage)	Study completion rates					✓
	Consultation and Relational Empathy (CARE) PREM (47)				✓	
	Measure Yourself Concerns and Wellbeing (MYCaW) PROM (40)			✓		
	Post-intervention qualitative questionnaire				✓	
	In-depth interviews with study participants				✓	
	Response quality/compliance with the validated outcome measures					✓
*To determine the optimal timing of therapy, measures of anxiety, worry, stress and self-management. Including intervention delivery and outcome measure compliance*						
Timing (Treatment utilisation)	Collecting data (dates) of when study participants utilised their massages				✓	
Anxiety	Generalized Anxiety Disorder Assessment (GAD-7) (43)	✓			✓	
Worry	Cambridge Worry Score (41)	✓			✓	
Stress	Perceived Stress Scale (46)	✓			✓	
Self-management	Revised Prenatal Coping Inventory (44) Strategies Used by People to Promote Health measure (45)	✓ ✓			✓	
*To conduct a thematic analysis of experiences of women undertaking the intervention to assess acceptability of the study and the intervention*						
Experience of the study intervention and study processes	Post-intervention qualitative questionnaire Consultation and Relational Empathy (CARE) PREM (47) In-depth interviews with study participants Measure Yourself Concerns and Wellbeing (MYCaW) PROM (40)				✓	
					✓	
					✓	
				✓		

^a^The measures used in this study may provide data for multiple project outcomes. “At the end of the study” is after the final participant has completed the intervention and completed the final post-study intervention questionnaires

#### Treatment utilisation

This data will describe when study participants utilised their massages (massage timing) to inform hypothesis generation regarding dosing value.

#### Validated self-reported questionnaires

Worry, anxiety, coping, self-efficacy, stress and empathy will be assessed in the study via the following questionnaires:

o Worry will be assessed via the *Cambridge Worry Score* (48) which is 17-item instrument that measures the content and extent of worries in pregnancy (48, 49). The total score range is from ‘zero to 85 with a higher score representing greater severity of worries’ (48).
o Maternal anxiety symptoms will be assessed *using the Generalized Anxiety Disorder Assessment (GAD-7)* (50) which is a 7-item instrument that measures the severity of generalised anxiety disorder. The total score range is from zero to 21 with scores of 5–9 representing mild anxiety, 10–14 moderate anxiety and 15–21 representing severe anxiety (50)*.*
o Coping via* the self-reported Revised Prenatal Coping Inventory (NuPCI)* (51) will be assessed. This scale has 32 items that evaluate the coping strategies of pregnant (51). The NuPCI consists of 3 subscales: positive attitude (15 items), avoidance (11 items) and spiritual-positive coping (6 items) (51). A higher score indicates more frequent use of a specific coping strategy (51).
o Self-efficacy via the *Strategies Used by People to Promote Health measure (SUPPH-29)* (52) will be assessed. This scale has 29 items that measure self-care self-efficacy (52). The SUPPH-29 consists of 3 sub-scales positive attitude (16 items), stress reduction (10 items) and decision making (3 items) (52). The total score range is from 29 to 145 with a higher score indicating greater self-confidence to carry out self-care strategies (52).
o Maternal stress symptoms via the *Perceived Stress Scale* (PSS) (53) will be assessed. The scale has 10 items that measure perceived personal stress (53). The total score range is from zero to 40 with a score ranging from 0 to 13 considered low stress, 14–26 moderate stress and 27–40 high stress (53).
o Empathy in the context of the therapeutic relationship via the *Consultation and Relational Empathy (CARE) patient-reported experience measure (PREM)* (54). This scale has 10 items that measures patients’ perceptions of relational empathy in the consultation (54). The total score range is from 10 to 40 with higher scores indicating greater perceived relational empathy (54).
o *Measure Yourself Concerns and Wellbeing (MYCaW)* assesses patient-reported outcomes *(PROM)* (46). This measure has the space for participants to write down two concerns they want help with, and then, the concerns are rated for severity using a 6-point Likert scale ranging from ‘not bothering me at all (0)’ to ‘bothers me greatly (6)’. A person’s wellbeing is also rated using the same 6-point Likert scale. There are two open-ended questions that ask if anything else important is happening in the person’s life and what has been most important about the treatment they received.

Researchers in the iCHOOSE Study are developing a core outcome set for stillbirth care research (55, 56), and this will guide future outcome measures for stillbirth research. The validated self-reported questionnaires evaluating worry and anxiety have been used together in previous research as outcome measures in stillbirth research (57) and influenced our use of these outcome measures. Worry, anxiety and stress have ‘intertwined behavioural and neural underpinnings’ (58); however, they have important unique characteristics that impact the way that they are experienced. Evaluating worry, stress and anxiety allows the researchers to capture the potential varied psychological states that can occur throughout a pregnancy after stillbirth especially when milestone events are passed without bad news/harm (e.g. gestation of previous loss, genetic and or anomaly scans).

*A post-intervention qualitative questionnaire* designed by the study researchers was based on a questionnaire used in previous stillbirth and massage research (36) (see Additional file 4) and assesses treatment utilisation and acceptability (Q.A-D), the appropriateness and suitability of the validated outcome measures the study used (Q.E), participant’s views of the study benefits (Q.G-L) and the participants’ perception of the study intervention to be a support for pregnant individuals via a massage intervention (Q.M).

An *in-depth interview* is intended to collect rich descriptions of participant experiences of massage and identify important aspects of care as well as provide information on the acceptability of the study intervention. Twenty study participants will be interviewed using a semi-structured approach by the study PI. The interviews will be conducted online via Zoom, recorded and transcribed verbatim by Cockatoo AI transcription services (59).

#### Data collection

Some women may give birth before receiving their allocated treatments. Intervention delivery and data collection will cease for participants who give birth prior to completing the full study protocol, and collected data will be kept within the data set. Attempts will be made to collect the final quantitative and qualitative data from participants who fall into this category. The inclusion criteria for participants to be no more than 30 weeks of gestation (Table 1) are in place to minimise participants giving birth prior to completing the full study protocol. Participants who give birth prior to completing the full study protocol will be emailed inviting them to complete the post-intervention online questionnaires and informing them that they are able to participate in the in-depth interview (if applicable) if they desire. A follow-up email will be sent 2 weeks after the first email.

## Data analysis

### Analysis of quantitative data

The participant’s demographic and clinical characteristics will be summarised using descriptive statistics. Summary statistics of the observational clinical treatment data (e.g. worry, anxiety, etc.) will be reported and an appropriate matched test will be used to determine significant differences between baseline and post-final treatment scores (e.g. paired *t*-test if data are normally distributed, Wilcoxon matched-pairs test if not).

Three treatment effect modifiers have been identified: (1) the amount of other support services utilised, (2) passing the gestational age during the study intervention that they experienced their stillbirth, (3) the time between the stillbirth and this pregnancy, and (4) the number of study intervention treatments received. The correlational analysis will be used to determine associations between observational treatment outcomes and support services utilized (e.g. talk therapy (psychology/counselling), peer support etc.). Appropriate comparative tests will be used to determine whether there is a relationship between observed treatment outcomes and gestation of stillbirth, the duration of the interpregnancy interval, and the number of treatments administered.

### Qualitative data analysis

The post-intervention questionnaire data and the open-ended MYCaW answers will undergo summative content analysis. The in-depth interview data will be analysed using thematic inductive analysis with the interview schedule modified to include emerging themes. Two researchers will immerse and familiarise themselves with the data to ascertain and identify the key concepts (60) The emerging themes will be discussed with members of the wider research team until a consensus is reached. An inductive approach will be utilised for analysis, as this method enables themes to be derived directly from the text data rather than being preconceived (60). To address biases in qualitative research methodology the researchers will provide a reflexivity statement acknowledging their role in the research and will undertake respondent validation which checks with participants to see if the findings still ring true.

There is considerable debate about what data saturation is and how it is determined depending on the qualitative research approach used and the data saturation model used (61–64). Instead of using the concept of data saturation as a determinate of qualitative data collection, the final sample size of our qualitative data collection will be shaped ‘by the adequacy (richness, complexity) of the data for addressing the research question’ (61) and the narrative approach that the final data will be presented as part of a mixed-methods methodology.

### Mixed-methods analysis

A convergent parallel mixed-methods methodology is utilised for the HOPES study. After separate analyses of the quantitative and qualitative data, the data will be compared and related for areas of convergence or divergence and then interpreted. A narrative and joint display approach will be used to integrate data, and the fit of data integration will be reported. A joint display is a visual framework and is intended to help compare findings from the qualitative and quantitative data and generate meta-inferences (65). A narrative approach is used to further develop and provide context for quantitative findings (66). The more vernacular feel of the narrative approach helps participating individuals ‘hear’ their voice in the findings.

### Dissemination of the findings

All participants will be offered a draft copy of any papers published as well as an easy-to-understand summary of the study findings. This will be emailed to the participants. A summary of the results and a copy of all the papers will be submitted to the funder.

## Discussion

Specialised antenatal care for women experiencing pregnancy after a stillbirth is recommended by researchers and clinicians (4, 13, 67); however, there is a lack of direct evidence on what specific interventions or approaches to care might benefit women experiencing pregnancy after stillbirth (15). The current research will begin to address the lack of high-grade evidence for supportive care options for individuals pregnant after a stillbirth. This protocol for a prospective feasibility study aims to measure the acceptability and preliminary effects of a massage intervention for women who are pregnant after a stillbirth or TMFR. The overarching intent of this work is to establish the feasibility of the protocol presented here for employment in a larger-scale intervention study. Progression criteria are listed below and use a Red-Amber-Green system based on previous feasibility work (14, 68) covering both qualitative and quantitative criteria (69).

### Feasibility success criteria

#### Progression criterion: Willingness of participants to use massage as a support option

Participants will be considered willing to consider using massage as a support option based on the recruitment of the sample.

- Red: Recruit < 60% of the required sample.
- Amber: Recruit 60–80% of the required sample.
- Green: Recruitment > 80%.

#### Progression criterion: Retention in the study

Participants will be considered retained in the study if they complete all the treatment intervention visits.

- Red: Retain < 60% of participants in the study.
- Amber: Retain 60–80% of participants in the study.
- Green: Retain > 80% of participants in the study.

#### Progression criteria: Intervention experienced as supportive

Participants will be considered to have found the intervention supportive if they answer yes to either ‘I feel very supported’ or ‘I feel extremely supported’ for Q.M in the post-intervention qualitative questionnaire.

- Red: Supportive < 60% of participants at the requisite level described above.
- Amber: Supportive 60–80% of participants at the requisite level described above.
- Green: Supportive > 80% of participants at the requisite level described above.

#### Progression criteria: Intervention implementation and experience

Assess the feasibility of delivering and experiencing the intervention in a way that is acceptable to pregnant women who have previously experienced stillbirth/TFMR.

- Red: Delivery and experience of intervention judged possibly feasible by qualitative data.
- Amber: Delivery and experience of intervention judged feasible by qualitative data.
- Green: Delivery and experience of intervention judged strongly feasible by qualitative data.

The methodology and design of the HOPES study use a model of integrating the participant as the expert on their needs for support and care during pregnancy after a stillbirth and reflects elements considered essential by families who have experienced a subsequent pregnancy after stillbirth. Specifically, Gower et al. found a high-quality excellent care involved affirming and recognising pregnant people’s anxiety while being kind and empathetic and incorporating individualized emotionally supportive care (4). This is reflected in the study design via the conceptual approach that massage is just more than the hands-on application of treatment and via the participants being able to make therapeutic decisions about what they require addressing at each treatment session as well as determining the timing of their treatments to meet their unique needs. A participant-centred, participant-led approach in clinical practice has been shown to improve satisfaction with the care experience and improve health outcomes (70, 71).

A key study strength is the application of the intervention in a real-world setting. This allows the researchers to analyse the potential effects and benefits of massage in a setting relatable to how massage is currently provided. This is important in translating any findings into practice and allowing commercial massage therapists to incorporate this type of massage into their practice. The sample selection bias due to participants self-selecting to participate in the study is a study limitation, but this sample is reflective of the clinical practice where consumers self-select to seek massage treatment. The lack of a comparison group may be considered a limitation of this study; however, the challenges of deploying an individual randomised-controlled trial design are recognised among researchers active in care in pregnancy after loss (15).

This paper outlines the research protocol for the HOPES study, a mixed-methods feasibility trial of a massage intervention for women who are pregnant after a stillbirth or TMFR. The overarching intent of this work is to establish the feasibility of the HOPES study protocol for employment in a larger-scale effectiveness trial.

## Data Availability

Not applicable.
