# Correction: Swedish massage as an adjunct approach to Help suppOrt individuals Pregnant after Experiencing a prior Stillbirth (HOPES): a convergent parallel mixed‑methods single‑arm feasibility trial protocol

**DOI:** 10.1186/s40814-024-01506-3

**Published:** 2024-05-11

**Authors:** Sarah Fogarty, Alexander E. P. Heazell, Niki Munk, Phillipa Hay

**Affiliations:** 1 School of Medicine, Western Sydney University, Locked Bag 1797, Penrith, NSW 2751 Australia; 2 Translational Health Research Institute, Western Sydney University, Locked Bag 1797, Penrith, NSW 2751 Australia; 3 School of Medical Sciences, Maternal and Fetal Health Research Centre, University of Manchester, Manchester, UK; 4 Department of Obstetrics, Saint Mary’s Hospital, Manchester University NHS Foundation Trust, Manchester, UK; 5 School of Health & Human Sciences, Indiana University, Indianapolis, USA; 6 Australian Research Centre in Complementary and Integrative Medicine (ARCCIM), Fellow and Visiting Faculty of Health, University of Technology Sydney, Massage & Myotherapy- Australia, Sydney, Australia; 7 Mental Health Services, SWSLHD, Campbelltown Hospital, Campbelltown, NSW Australia

**Correction: Pilot Feasib Stud 10, 67 (2024)**

**https://doi.org/10.1186/s40814-024-01499-z**

Following publication of the original article [1], the authors reported an error to the affiliations of Sarah Fogarty and Alexander E.P Heazell. Affiliation 4 “Department of Obstetrics, Saint Mary’s Hospital, Manchester University NHS Foundation Trust, Manchester, UK” should be removed from Sarah Fogarty and added to Alexander E.P. Heazell and affiliation 2 “Translational Health Research Institute, Western Sydney University, Locked Bag 1797, Penrith, NSW 2751, Australia “ should be removed from Alexander E.P Heazell.

The original article [1] has been updated.
